# A Primer on Python for Life Science Researchers

**DOI:** 10.1371/journal.pcbi.0030199

**Published:** 2007-11-30

**Authors:** Sebastian Bassi

**Affiliations:** Whitehead Institute, United States of America

## Introduction

This article introduces the world of the Python computer language. It is assumed that readers have some previous programming experience in at least one computer language and are familiar with basic concepts such as data types, flow control, and functions.

Python can be used to solve several problems that research laboratories face almost everyday. Data manipulation, biological data retrieval and parsing, automation, and simulation of biological problems are some of the tasks that can be performed in an effective way with computers and a suitable programming language.

The purpose of this tutorial is to provide a bird's-eye view of the Python language, showing the basics of the language and the capabilities it offers. Main data structures and flow control statements are presented. After these basic concepts, topics such as file access, functions, and modules are covered in more detail. Finally, Biopython, a collection of tools for computational molecular biology, is introduced and its use shown with two scripts. For more advanced topics in Python, there are references at the end.

## Features of Python

Python is a modern programming language developed in the early 1990s by Guido van Rossum [1]. It is a dynamic high-level language with an easily readable syntax. Python programs are interpreted, meaning that there is no need for compilation into a binary form before executing the programs. This makes Python programs a little slower than programs written in a compiled language, but at current computer speeds and for most tasks this is not an issue and the portability that Python gains as a result of being interpreted is a worthwhile tradeoff.

The more important and relevant features of Python for our use are that: it is easy to learn, easy to read, interpreted, and multiplatform (Python programs run on most operating systems); it offers free access to source code; internal and external libraries are available; and it has a supportive Internet community.

Python is an excellent choice as a learning language [2]. The language's simple syntax uses mandatory indentation and looks similar to the pseudocode found in textbooks that are oriented to non-programming students. Its simplicity is a design choice, made in order to facilitate the learning and use of the language. Another advantage well-suited to newcomers is the optional interactive mode that gives immediate feedback of each statement, which certainly encourages experimentation.

There are also some drawbacks to Python that must be noted. First, execution time is slower than for compiled languages. Second, there are fewer numerical and statistical functions available than in specialized tools like R or MATLAB. (However, Numpy module [3] provides several numeric and matrix manipulation functions for Python.) And third, Python is not as widely used as JAVA, C, or Perl.

## Tutorial

### 

#### Notations.

Program functions and reserved words are written in **bold** type, while user-defined names are in *italics.* For computer code, a monospaced font is used. Three angle braces (>>>) are used to indicate that a command should be executed in the Python interactive console. The line shown after the user-typed command is the result of that command.

#### The absolute basics.

Python can be run in script mode (like C and Perl), or using its built-in interactive console (like R and Ruby). The interactive console provides command-line editing and command history, although some implementations vary in features. In the interactive mode, there is a command prompt consisting of three angle braces (>>>).

Script mode is a reliable and repeatable approach to running most tasks. Input file names, parameter values, and code version numbers should be included within a script, allowing a task to be repeated. Output can be directed to a log file for storage. The interactive console is used mostly for small tasks and testing.

Python programs can be written using any general purpose text editor, such as Emacs or Kate. The latter provides color-cued syntax and access to Python's interactive mode through an integrated shell. There are also specialized editors such as PythonWin, Eclipse, and IDLE, the built-in Python text editor.

When running a Python script under a Unix operating system, the first line should start with “#!” plus the path to the Python interpreter, such as “#!/usr/bin/python”, to indicate to the UNIX shell which interpreter to employ for the script. Without this line, the program will not run from the command line and must be called by using the interpreter (for example, “python myprogram.py”).

**Table 1:**
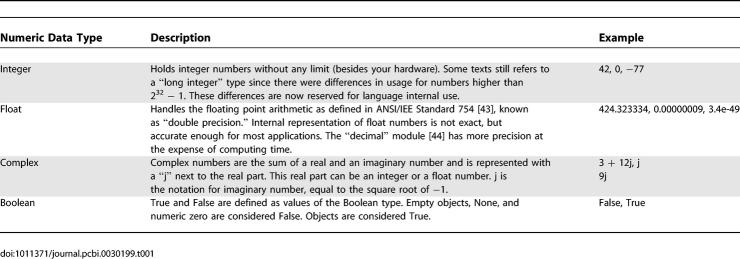
Numeric Data Types

Computer languages can be characterized by their data structures (or types) and flow control statements. Data structures in Python are diverse and versatile. There are numeric data types that hold “primitive” data (integer, float, Boolean, and complex) and there are “collection” types that can handle several objects at once (string, list, tuple, set, and dictionary). Descriptions and examples of numeric data types are summarized in Table 1. Container types can be divided according to how their elements are accessed. When ordered, they are sequence types (string, list, and tuple being the most prominent). Sequence types can be accessed in a given order, using an index. Their differences, methods, and properties are summarized in Box 1. There are also unordered types (sets and dictionaries). Both unordered types are described in Box 2.

**Box 1. Most-Used Sequence Data Types**

**String:**

Usually enclosed by quotes (') or double quotes ("). Triple quotes (''') are used to delimit multiline strings. Strings are immutable. Once created they can't be modified. String methods are available at http://www.python.org/doc/2.5/lib/string-methods.html.

For example:

>>> s0='A regular string'

**List:**

Defined as an ordered collection of objects; a versatile and useful data type. C programmers will find lists similar to vectors. Lists are created by enclosing their comma-separated items in square brackets, and can contain different objects.

For example:

>>> MyList=[3,99,12,"one","five"]

This statement creates a list with five elements (three numbers and two strings) and binds it to the name “MyList”. Each element of the list can be referred to by an integer index enclosed between square brackets. The index starts from 0, therefore MyList[3] returns “one”. All list operations are available at http://www.python.org/doc/2.5/lib/typesseq-mutable.html.

**Tuple:**

Also an ordered collection of objects, but tuples, unlike lists, are immutable. They share most methods with lists, but only those that don't change the elements inside the tuple. Attempting to change a tuple raises an exception. Tuples are created by enclosing their comma-separated items between parentheses. Tuples are similar to Pascal records or C structs; they are small collections of related data that are operated on as a group. They are used mostly for encapsulating function arguments, or any data that are tightly coupled.

For example:

>>> MyTuple=(2,3,10)

Tuple operations are available at http://www.python.org/doc/2.5/lib/typesseq.html.

**Box 2. Unordered Types**

**Set:**

An unordered collection of immutable values. It is mostly used for membership testing and removing duplicates from a sequence. Sets are created by passing any sequential object to the set constructor, such as: set([1,2,3])

For more information on sets, please refer to http://www.python.org/doc/2.5/lib/types-set.html.

For example:

>>> ResEzSet1=set(['BamH1', 'HindIII', 'EcoR1', 'SalI'])

>>> ResEzSet2=set(['PlaA', 'EcoR1', 'Eco143'])

>>> ResEzSet1&ResEzSet2

set(['EcoR1'])

**Dictionary:**

A data type that stores unordered one-to-one relationships between keys and values. Unordered in this context means that each key–value pair is stored without any particular order in the dictionary. It is analogous to a hash in Perl or a Hashtable class in Java. Dictionaries are created by placing a comma-separated list of key–value pairs within braces.

For example:

Set Translate as a dictionary with codon triplets as keys and the corresponding amino acids as values:

>>> Translate={"cca":"P","cag":"Q","agg":"R"}

Creating a new entry:

>>> Translate["gat"]="D"

To see what is inside the dictionary:

>>> Translate

{'agg': 'R', 'cag': 'Q', 'gat': 'D', 'cca': 'P'}

Dictionaries share some methods with lists. A complete list of methods on can be seen at: http://www.python.org/doc/2.5/lib/typesmapping.html.

Flow control statements control whether program code is executed or not, or executed many times in a loop based on a conditional. Conditional execution (**if**, **elif**, **else**) and looping (**for** and **while**) are explained in Box 3.

**Box 3. Control Structures**

**If statement:**

Tests for a condition and acts upon the result of that condition. If the condition is true, the block of code after the “if condition” will be executed. If it is false, the program will skip that block and will test for the next condition (if any). Several conditions can be tested using **elif**. If all conditions are false, the block under **else** will be executed. **Elif** can be used to emulate a C “switch-case” statement.

Scheme of an if statement:

**if** condition1:

 block1

**elif** condition2:

 block2

**else**:

 block3

**For loop:**

Iterates over all the members of a sequence of values (as in Perl's “foreach”). It is different from C and VB because there isn't a variable that increments or decrements on each cycle. This sequence could be any type of iterable object like a list, string, tuple, or dictionary. The code inside a for loop will be executed once for each item in the sequence, and at the same time the variable will take the value of each item in the sequence. There could be an optional **else** clause. If it is present, the block under the else clause is executed when the loop terminates through exhaustion of the list, but not when the loop is terminated by a **break** statement.

The structure of a for loop is:

**for** variable **in** sequence:

 block1

**else**:

 block2

**while loop:**

Executes a block of code as long as a condition is true. As the for loop, there could be an optional **else** clause. When present, the block under the else clause is executed when the condition becomes false but not when the loop is terminated by a **break** statement.

The general form is:

**while** condition:

 block1

**else**:

 block2

#### Functions.

Python allows programmers to define their own functions. The **def** keyword is used followed by the name of the function and a list of comma-separated arguments between parentheses. Parentheses are mandatory, even for functions without arguments.

Python's function structure is:

**def** FunctionName(argument1, argument2, ...):

*function_block*

**return** *value*

Arguments are passed by reference and without specifying data types. It is up the programmer to check data types. When a function is called, the arguments must be supplied in the same order as defined, unless arguments are provided by using keyword–value pairs (*keyword=value*). Default arguments can be defined by using keyword–value pairs in the function definition. This way a function can be called without supplying arguments. To deal with an arbitrary number of arguments, the last argument in the function definition must be preceded with an asterisk in the form *name. This specifies that the last value is set to a tuple for all remaining parameters.

The return statement terminates the execution of the function and returns a single value. To return multiple values, a list or a tuple must be used.

#### Modules.

In Python, functions, classes and constants can be saved in a file, called a “module,” for later use. Modules can be called from a program or in interactive mode using the “import” statement, such as:

**import** ModuleName

where ModuleName is the name of the file without an extension. When a module is imported for the first time, its code is interpreted and executed. Execution upon import of certain code can be prevented by putting the code into an import executable conditional statement (if __name__ == __main__). The '__name__' attribute of the module is the name of the module and is '__main__' only when the module is run as a standalone program. Successive imports of the same module have no effect.

Python provides several modules and there are many more that can be downloaded from the Internet (like SciPy [4], which provides scientific and numeric tools for Python, Matplotlib [5] for plotting, and so on).

An example:

>>> **import** math

>>> **dir**(math)

['__doc__', '__file__', '__name__', 'acos', 'asin', 'atan', 'atan2', 'ceil', 'cos', 'cosh', 'degrees', 'e', 'exp', 'fabs', 'floor', 'fmod', 'frexp', 'hypot', 'ldexp', 'log', 'log10', 'modf', 'pi', 'pow', 'radians', 'sin', 'sinh', 'sqrt', 'tan', 'tanh']

No error message returned by the interpreter means that the module was successfully imported. **dir()** is a built-in function that returns a list of the attributes and methods of any chosen object. To access an object from a module, the syntax is module.object, since importing a module creates a namespace.

For example:

>>> math.log(2)

0.69314718055994529

## Python in Action: Net Charge from an Amino Acid Sequence

To show Python syntax and data structures in action, it is instructive to look at solving a real problem using this language, such as the calculation of the net charge of a protein. Given a protein sequence, this is performed by adding up the charges of each charged amino acid at pH = 7. This calculation gives a rough value because it doesn't consider whether the residues are exposed, partly exposed, buried, or deeply buried. This example shows functions, data types (numbers, strings, and dictionaries) and flow control (**if** and **for**).

### 

#### Code explained.

This script defines a function (*netcharge*) that takes a peptide sequence as an input (*seq*) and calculates the net charge by adding up all the individual charged amino acids. The main data structure is a dictionary (*AACharge*) with the values of each charged amino acid. There is also a numeric type (*charge*) that holds the partial charge values and is initialized with −0.002 since this is the value of the net charge of the amino and carboxy-terminus of the peptide. For each amino acid, the program checks whether it is inside the list of the keys in the dictionary and, if it is, adds its charge values. After the function definition, a protein sequence is called as an argument. The commented source code is shown in Protocol S1.

#### Biopython.

Biopython is a distributed, collaborative effort to develop Python libraries and applications that address the needs of current and future work in bioinformatics [6]. It provides tools for working with biological sequences, parsers of popular file formats used in bioinformatics (FASTA, COMPASS [7], GenBank, PIR [8], PDB [9], BLAST output [10], InterPro [11], LocusLink [12], PROSITE [13], Phred [14], Phrap [15]), data retrieval from biological databases (Swiss-Prot [16], PubMed [17], GenBank [18]), a wrapper for bioinformatics programs (BLAST, ClustalW [19], EMBOSS [20], Primer3 [21], and more), functions to estimate DNA and protein properties such as isoelectric points [22,23], restriction enzymes cutting, and many more.

A review of Biopython functions would require a far more considerable amount of space; therefore this paper shows only a small portion of the bigger picture. The first example shows how to parse a BLAST output to extract and report only required features. Since BLAST is the most commonly used application in bioinformatics, writing a BLAST report parser is a basic exercise in bioinformatics [24]. Other functions like massive file processing and file format conversion are also shown.

## Parsing BLAST Files

The program below extracts the title and sequence from some high-scoring pairs (HSP), but there are many more features to extract from a BLAST output, if needed. Biopython provides the Blast Record class under Bio.Blast.NCBIXML.Record. Internal documentation for this object can be accessed with **help**(NCBIXML.Record) after importing NCBIXML from Bio.Blast.

### 

#### Code explained.

For this program, the user has to perform a BLAST search and save the result in XML mode because this format tends to be more stable than HTML or text versions (and hence the Biopython parser should be able to handle it without any problem [25]). The BLAST search can be performed using the NCBI Web server (http://www.ncbi.nlm.nih.gov/BLAST/). To generate XML output, select XML as the format option on the BLAST page. When using the standalone version of BLAST, the *m* parameter in the blastall command should be set to 7. Biopython can also be used to run the BLAST program; in this case the output defaults to XML. A sample XML BLAST output (Blast2.xml) is provided in Protocol S4. The objective is to print out only the sequences with an HSP larger than 80 base pairs in a specific chromosome.

This program, blastparser2.py, takes a BLAST output in XML format and shows the sequence of hits in Chromosome 5 that are larger than 80 base pairs long. A file handle named bout with the BLAST output in XML format is created, and then the file is parsed using the **Bio.Blast.NCBIXML.parse** function. To parse another type of BLAST output, the parser should be changed. Instead of using NCBIXML, use NCBIStandalone. All BLAST records are stored in an iterator called *b_records*. Using a **for** loop, the program steps through all the BLAST records. For each hit, the program checks all HSP for the presence of the “chromosome 5” string and a length of the hit sequence (without gaps) greater than 80 base pairs. The source code is shown in Protocol S2.

## Consolidate Several Sequence Files into One FASTA File

In this example we use a simulated output provided by an external sequencing service. It consists of more than 6,000 directories (one for each clone), and there are three files per directory (a formatted report with a pdf extension, the sequencing machine output with an ab1 extension, and a plain text file with the sequence). This directory structure and its files are available as Protocol S4. The program retrieves all the sequences and writes them into a FASTA-formatted file for further analysis.

The program fromdir2fasta.py to scan a directory (mydir) where the output of the sequencing service is downloaded. The names of all the directories under that directory are obtained with the **os.listdir** function and stored into a list (*lsdir*). For each directory (*x*), the list of files are stored into a list (*fs*). For each file (*curfile*), the program checks if it ends in “txt,” and if it does the script retrieves the sequence from the file as a Seq object (*dna*). Using the title from the filename and the seq object, it creates a SeqRecord object (seq_rec) and adds it to a list (*sequences*). After the directory had been scanned and the sequences list filled with SeqRecord objects corresponding to all the files, the sequences are written to a file in FASTA format. This is done with the **SeqIO.write** function. For an explanation of file handling, see Box 4. To get the output in another format, the third parameter of this function should be changed. For more information on SeqIO, including a table with supported formats, see http://biopython.org/wiki/SeqIO. The commented source code is shown in Protocol S3.

**Box 4. Dealing with Text Files**

Reading a text file in Python is a three-step process.

1: Open the file, creating a handle.

handle=**open**('PathToFile','r')

The first parameter is the filename location. The second parameter is the first letter of the open mode, that is, r, w, and a, corresponding to read, write, and append. This function returns a file object (handle).

2: Read the file. There are several methods to gain access to the contents of a file:

handle.**read**(n): Reads the first *n* bytes of a file and returns a string. Without arguments, reads the file until the end of file (EOF).

handle.**readline**(n): Reads a line of the file and returns a string. When it reaches the EOF, it returns an empty string.

handle.**readlines**(): Reads all the lines and returns a list of strings. The “end of line” (EOL) is determined based on host operating system.

For efficient iteration over a file, use “**for** line **in** handle”.

3: Close the file:

handle.**close**(): Closes the file.

Writing a file is very similar to reading a file. Steps 1 and 3 are the same as reading a file. The main difference is in step 2, where the file's contents are written with the **write** method, as:

handle.write(“This text will make it into a text file\n”)

There is also a writelines method that writes each member of the list to a file.

## Summary

Python's capabilities include scientific plotting [5,26–29], GUI building [30–32], automatic Web page generation [33–35], and interfacing with Windows components [36,37] and with external programs like R [38] and Matlab [39]. As hardware becomes faster, a computer's raw processing time is less relevant than scientist's time [40]. Scripting languages allow the programmer to do more in less time, making Python an excellent choice for bioinformatics data analysis.

## Additional Reading

One common problem for non-computer science researchers who start programming is that they usually stick to basic concepts and don't take advantage of many modern tools that are available [41]. Version control, project management, and automatic unit testing are only a handful of useful software engineering techniques that are virtually unknown to most researchers [42].

There are many good quality resources for learning Python. Some of these have already been mentioned and a summary of resources is presented in Table 2. Since code written in Python is easy to read, modifying other people's code to suit your needs is a recommended path for learning. For this reason, the table also includes code search engines and bioinformatics software repositories.

**Table 2:**
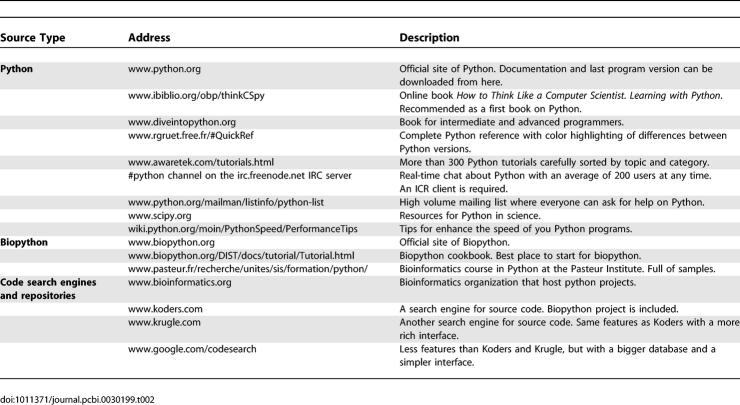
Resources for Learning Python and Biopython

## Supporting Information

Protocol S1 This Program Defines a Function To Calculate the Net Charge of a Protein Based on the Charges of Its Amino Acids On the last line of the code the function is called.
(107 KB DOC)

Protocol S2 This Program Reads the Output of a BLAST Run Using the Parse Function on the NCBIXML Module (107 KB DOC)

Protocol S3 This Program Shows How To Use Python to Mass-Convert Sequence Files from Plain Text to FASTA Format with Biopython SeqIO Module (48 KB DOC)

Protocol S4 Python Code and Needed Files To Run Programs (172 KB GZ).

## Abbreviations

HSP: high-scoring pairs
