# Supplementary material for: A Primer on Python for Life Science Researchers
Source: PLoS Comput Biol. 2007 Nov 30;3(11):e199. doi: 10.1371/journal.pcbi.0030199 (PMC2098836; doi:10.1371/journal.pcbi.0030199)
Supplement: Protocol S1 — On the last line of the code the function is called. (107 KB DOC) [file pcbi.0030199.sd001.doc]

| Python source code | Comments |
| --- | --- |
| **def** netcharge(seq): | Define the function (netcharge) that takes a string (seq) as input |
| charge = -0.002 | Set a starting value for the amino acid charge (charge) |
| AACharge={"C":-.045,"D":-.999,"E":-.998,"H":.091\  ,"K":1,"R":1,"Y":-.001} | Set a dictionary (AACharge) with the values of net charge for each charged amino acid. |
| **for** aa **in** seq: | For each amino acid in the input sequence |
| **if** aa **in** AACharge: | Check if the amino acid is present in the dictionary |
| charge=charge+AACharge[aa] | Add to charge the value of the charged amino acid |
| **else**: |  |
| **pass** | Do nothing. This is an optional statement used to make the code clear |
| **return** charge | Return the sum of all charged amino acids (charge) |
| **print** netcharge('MEFDPTKINISSIDHVTILQYIDEPND') | Call the function with a sequence as parameter and print to stdout. |
