# Supplementary material for: A Primer on Python for Life Science Researchers
Source: PLoS Comput Biol. 2007 Nov 30;3(11):e199. doi: 10.1371/journal.pcbi.0030199 (PMC2098836; doi:10.1371/journal.pcbi.0030199)
Supplement: Protocol S2 — (107 KB DOC) [file pcbi.0030199.sd002.doc]

| Python source code | Comments |
| --- | --- |
| **from** Bio.Blast **import** NCBIXML | Import NCBIXML module |
| bout=**open**('Blast2.xml') | Filehandle (*bout*) for input file (Blast2.xml). See Box 4 for information on file handling |
| b_records=NCBIXML.parse(bout) | Parse *bout* file and bind the output to the name *b_records* |
| **for** b_record **in** b_records: | For each Blast record (*b_record*) in all the records (*b_records*) |
| **for** alig **in** b_record.alignments: | For each aligment object (*alig*) in the list of alignments |
| **for** hsp **in** alig.hsps: | For each hsp object (*hsp*) in the current alignment |
| hitseq=hsp.sbjct.replace('-','') | Remove gap characters (-) from hsp and keep the ungapped sequence (*hitseq*) |
| **if** "chromosome 5" **in** alig.title \  **and** len(hitseq)>80: | Check if “chromosome 5” is in the title of the alignment and if the length of *hitseq* es greater than 80 basepairs long |
| **print** alig.title+"\t"+hitseq | Print the results separated by tabs |
