# Supplementary material for: A Primer on Python for Life Science Researchers
Source: PLoS Comput Biol. 2007 Nov 30;3(11):e199. doi: 10.1371/journal.pcbi.0030199 (PMC2098836; doi:10.1371/journal.pcbi.0030199)
Supplement: Protocol S3 — (48 KB DOC) [file pcbi.0030199.sd003.doc]

| Python source code | Comments |
| --- | --- |
| **import** os | Import os module |
| **from** Bio **import** SeqIO | Import SeqIO module |
| **from** Bio.Seq **import** Seq | Import Seq class |
| **from** Bio.SeqRecord **import** SeqRecord | Import SeqRecord class |
| lsdir=os.listdir('mydir') | Creates a list with all the directory names under “mydir” directory and name it lsdir |
| outf=**open**('combined.fasta','w') | Set a filehandle (outf) for the output file (combined.fasta). For information on file management, see Box 4. |
| sequences=[] | Initialize an empty list (sequences) |
| **for** x **in** lsdir: | For each directory name (x) in the list of directory names (lsdir) |
| fs=os.listdir('mydir/'+x) | List of the filenames under “mydir” and store them in a list (fs) |
| **for** curfile **in** fs: | For each filename (curfile) in the list of filenames (fs) |
| **if** curfile[-3:]=='txt': | Check if the filename ends in txt |
| txtfile=**open**('mydir/'+x+'/'+curfile) | Filehandle (txtfile) for current text file. See Box 4 for information on file handling |
| dna=Seq(txtfile.readline()) | Set a Seq object (dna) with the sequence inside the text file |
| seq_rec=SeqRecord(dna,curfile[:-4],'','') | Set a SeqRecord object with dna sequence and the name of the text file without the extension |
| txtfile.**close**() | Close the text file |
| sequences.append(seq_rec) | Add the newly created SeqRecord object into a list (sequences) |
| SeqIO.write(sequences,outf,'fasta') | Write all the SeqRecord objects in the sequences list into the output file (outf)in fasta format |
| outf.**close**() | Close the output file |
